# Modeling and simulation of heat production in a local heating installation including a batch-fired straw boiler with a buffer tank

**DOI:** 10.1016/j.heliyon.2020.e05667

**Published:** 2020-12-07

**Authors:** Wojciech Kreft

**Affiliations:** AGH University of Science and Technology, Faculty of Electrical Engineering, Automatics, Computer Science and Biomedical Engineering, Department of Automatic Control and Robotics, Al. Mickiewicza 30, 30-059 Krakow, Poland

**Keywords:** Applied mathematics, Computational mathematics, Energy, Thermodynamics, Computational heat transfer, Mathematical modeling, Systems theory, Batch-fired straw boiler, Biomass, Combustion, Modeling, Model identification, Renewable energy sources

## Abstract

The paper presents the problem of modeling and simulation of thermal energy generation in a batch-fired straw boiler combined with a buffer tank. The batch-fired straw boiler in concern have a combustion chamber and a water jacket. During the combustion process, the combustion chamber heats up and from it the water jacket. Within the boiler, water circulates between the water jacket and the buffer tank.

The author proposes a thermal model of the heating installation and a method of identifying the parameters of this model.

This model has been simulated in the MATLAB/Simulink environment and presented as an electric analogue.

The model of the system has been validated, which means that system parameters have been identified. This identification was based on the results of measurements of the straw combustion processes which were conducted using a laboratory installation. Finally, a model consistent with actual experiments was obtained. The presented model allows for the observation of changes in heating power consumption variations occurring during the straw feed combustion, which depend on the operating parameters of the system. The results of the model can be applied in the optimization of the configuration of the installation.

## 1 Introduction

Over the last decades, the issues of environmental protection have been raising increasing interest. The most important problems include the hazards caused by CO_2_ emissions generated in energy conversion processes [1, 2, 3, 4]. The international community makes significant efforts to reduce the global emissions of greenhouse gases. One of the first steps taken in this direction was the Kyoto Protocol [5]. The latest of the significant international documents concerning the prevention of global warming include the “Post-2020 climate change agreement”, announced at COP 21 in Paris at the end of the 2015 [6].

The International Energy Agency recognized that the growing use of renewable energy sources is a vital factor in tackling global climate changes. Renewable energy technologies are advantageous not only due to the resulting reduction of CO_2_ emissions. The benefits also consist in the creation of workplaces, promotion of comprehensive regional development and improvement in energy security.

According to the IEA standpoint, biomass-derived products are considered renewable energy sources. Thus, biomass is an organic matter that may be converted to fuel.

Provided that the combustion process is properly managed, biomass combustion in special boilers does not pollute the environment. Therefore, the operation of these boilers takes into account control of ecological and strictly energy issues [7, 8, 9].

Straw constitutes a particular type of biomass, which is a by-product of cereal production. The production of straw for energy purposes is an important contribution to the development of renewable energy sources. In Poland, for example, the total surplus of cereal straw which may be used for energy purposes amounts to approximately 12.7 million tons per year [10].

Straw combustion for water heating purposes is carried out in specially designed boilers which serve the heating of residential and non-residential buildings [11, 12].

Simple straw boilers are the so-called batch-fired straw boilers. The nominal thermal power of such boilers is about 50–500 kW [13]. The straw is fed to these boilers in batches. Straw batches are usually in the form of pressed bales. The combustion process ends with a complete combustion of the straw bale. Thus, the batch-fired straw boiler operates in cycles. As a result, there are significant variations of the water temperature at the boiler outlet. To eliminate boiler short circuiting, a proper buffer tank should be used. Owing to the buffer tank, hot water may be continuously supplied to the radiators even in case of interrupted boiler operation.

This paper concerns the mathematical model of thermal energy generation in a local heating installation. The thermal processes in a batch-fired straw boiler combined with a buffer tank have been modeled. The novelty of this paper is constituted by the proposed model of the entire installation. This model has been verified using experiments of straw combustion in the installation located at the laboratory of the AGH University of Science and Technology. As a result of the verification, slight changes have been introduced to the straw combustion model. This issue concerned the change of the straw burning rate factor over time. According to the best knowledge of the author, this approach to modeling the combustion process and some other parts of the entire model has not been presented so far.

The results of the simulations have been compared with the combustion experiments. Comparison the model with the experiments led to the validation of the model (identification of its parameters). While initially the combustion process model proved to be incompatible with the experimental results, after its slight modification it proved to be exceptionally compatible with the experimental results.

This model proves useful because it does not require analysis of the chemical reactions occurring during combustion, but only the analysis of thermal energy generation from the unit of straw mass and the rate of its combustion.

## 2 Process of straw combustion in a batch-fired boiler

**Figure 1:**
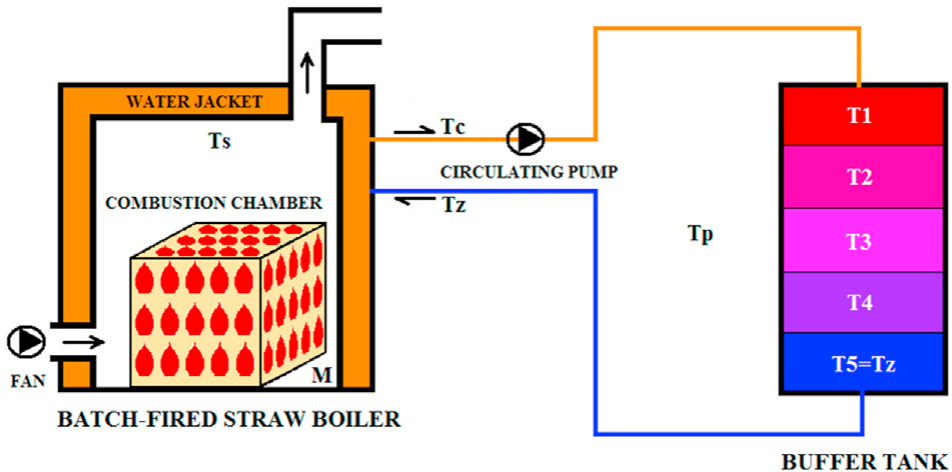
Schematic diagram of the boiler with a buffer tank.

**Figure 2:**
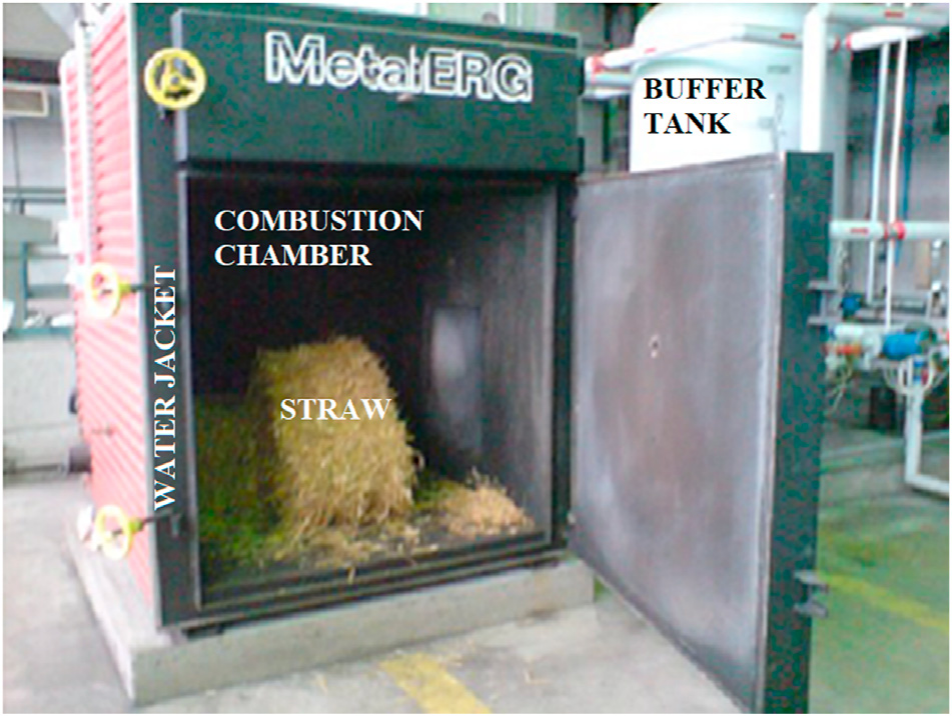
Heating installation at the AGH University of Science and Technology.

The analyzed installation includes a straw boiler and a buffer tank (Figure 1 and Figure 2). The main part of the boiler is a combustion chamber where the combustion process takes place. Around the combustion chamber there is a water jacket which is filled with water. The pressed straw is fed into the combustion chamber and is set on fire through a special tube. The air is blown into the combustion chamber by a fan. Within the system, water – forced by a circulating pump – continuously circulates between the boiler and the buffer tank. Water heated in the jacket is pumped to the buffer tank and returns to the jacket as cooled down.

The straw combustion process may be modeled in several manners [14, 15, 16, 17, 18]. A detailed analysis of the combustion process requires taking into account physical and chemical phenomena. The physical processes are the heat and mass transfers, while the chemical processes are constituted by chemical reactions proceeding during the straw combustion. Chemical reactions occurring during combustion may, for example, be described with the assumption that the main components of the straw as a kind of biomass are constituted by carbon, hydrogen and oxygen, expressed as the molar fractions thereof. Assuming that the CO and NO_x_ compounds in the exhaust gas are negligible and that the combustion has been complete, a reaction scheme for combustion may be represented by Eq. (1) [19].

(1) $$C_{z}H_{y}O_{x}+r\left(z+\frac{y}{4}-\frac{x}{2}\right)O_{2}+r\frac{79}{21}\left(z+\frac{y}{4}-\frac{x}{2}\right)N_{2}\to zCO_{2}+\frac{y}{2}H_{2}O+r\frac{79}{21}\left(z+\frac{y}{4}-\frac{x}{2}\right)N_{2}+\left(r-1\right)\left(z+\frac{y}{4}-\frac{x}{2}\right)O_{2}$$

In the equation, *x*, *y*, and *z* represent the molar fractions of oxygen, hydrogen and carbon, respectively, in one mole of the fuel, while r represents the air excess factor in the combustion. The elemental composition of the combustion products together with the type of process and the characteristics of the reactor determine the mechanisms causing the formation of by-products. Thus, the by-products depend on the concentrations of reactants and temperature of combustion.

In case of physical processes occurring during the combustion in the batch-fired straw boiler, it should be noted that the spatial temperature is not uniform within the combustion chamber [20]. Within the chamber, there are some locations covered by flames as well as ones subject to a higher airflow.

During straw combustion, thermal energy is released to the combustion chamber and there is a progressive conversion of the straw mass into gas and ash.

The models usually do not need to precisely describe the physical and chemical processes but it is sufficient to take the most important effects into account. To achieve that, the static and dynamic features of the system should be analyzed. The straw combustion model presented below is based on the author's earlier papers [21, 22].

Analyzing the burning straw, the mass flow of gases discharged to the chimney equals the sum of the mass flows of the air forced by the fan and volatile substances from the burning straw. The combustion rate depends on the air flow and the kind of feed material. The combustion itself takes place at the surface of the straw lump, because only at this surface straw and air coexist, which is necessary to sustain combustion. The change in burning mass can be described by a differential Eq. (2). It is assumed that the combustion rate at a given point is proportional to the current surface area subject to combustion, the air mass flow and the burning rate factor, which is specified for particular types of straw. It is also assumed that the straw fed to the chamber is cube-shaped, and it maintains this shape during the whole burning time. The mass and volume of the ash generated during straw combustion have been neglected.

(2) $$\frac{d}{dt}M_{s}\left(t\right)=-5\alpha F_{p}\left(t\right){\left(\frac{M_{s}\left(t\right)}{\rho_{s}}\right)}^{\frac{2}{3}}$$

where:

*t* – time [s],
*M*_*s*_(*t*) – straw mass [kg],
*F*_*p*_(*t*) – air mass flow [kg/s],
*ρ*_*s*_ – straw density [kg/m^3^],
*α* – straw burning rate factor [1/m^2^].

## 3 Comprehensive model of the installation

An accurate description of the analyzed system is a complex issue. The problem is related not only to the changes of the variables in time, but also in space. This problem may be solved by using the Finite Element Method [23, 24, 25, 26]. It is, however, possible to describe the thermal processes less accurately, but in a much simpler manner. This approach assumes the uniformity of temperatures in particular regions [27, 28] leading to the so-called lumped-parameter system. In case of the problem described in this section, temperatures of the combustion chamber, each boiler wall and the water jacket are considered uniform.

Using Eq. (2) one can build a thermal model of the analyzed installation. This model can be presented by Eqs. (3), (4), (5), (6), (7), (8), (9), (10), and (11). This model assumes that heat is transferred by conduction (e.g. boiler walls – water jacket), convection (e.g. combustion chamber – inner walls, burning straw – combustion chamber) and radiation (burning straw also heats the boiler's inner walls directly).

(3–11) $$\left\{ \begin{array}{l} \frac{d}{dt}\left(V_{k}\left(t\right)\rho_{sp}\left(t\right)+M_{s}\left(t\right)\right)=\;F_{p}-F_{sp}\left(t\right)\\ c_{sp}\frac{d}{dt}\left(M_{s}\left(t\right)T_{sp}\left(t\right)+V_{k}\left(t\right)\rho_{sp}\left(t\right)T_{sp}\left(t\right)\right)=P\left(t\right)\beta +F_{p}c_{p}T_{p}\left(t\right)-F_{sp}\left(t\right)c_{sp}T_{sp}\left(t\right)-K_{s\_sc}\left(T_{sp}\left(t\right)-T_{sc}\left(t\right)\right)\\ M_{sc}c_{sc}\frac{d}{dt}T_{sc}\left(t\right)=K_{s\_sc}\left(T_{sp}\left(t\right)-T_{sc}\left(t\right)\right)+P\left(t\right)\left(1-\beta \right)+K_{w\_sc}\left(T_{c}\left(t\right)-T_{sc}\left(t\right)\right)\\ M_{w}c_{w}\frac{d}{dt}T_{c}\left(t\right)=F_{w}c_{w}\left(T_{z}\left(t\right)-T_{c}\left(t\right)\right)+K_{w\_sc}\left(T_{sc}\left(t\right)-T_{c}\left(t\right)\right)+K_{w\_sc}\left(T_{sc\_zew}\left(t\right)-T_{c}\left(t\right)\right)\\ M_{sc\_zew}c_{sc}\frac{d}{dt}T_{sc\_zew}\left(t\right)=K_{w\_sc}\left(T_{c}\left(t\right)-T_{sc\_zew}\left(t\right)\right)+K_{p\_sc\_zew}\left(T_{p}\left(t\right)-T_{sc\_zew}\left(t\right)\right)\\ \frac{\mu p}{R}=\rho_{sp}\left(t\right)T_{s}\left(t\right)\\ V_{k}\left(t\right)=V_{0}-\frac{M_{s}\left(t\right)}{\rho_{s}}\\ \frac{d}{dt}M_{s}\left(t\right)=-5\alpha F_{p}{\left(\frac{M_{s}\left(t\right)}{\rho_{s}}\right)}^{\frac{2}{3}}1\left(t-\theta \right)\\ -\lambda \frac{d}{dt}M_{s}\left(t\right)=P\left(t\right) \end{array}\right.$$

where:

*t* – time [s],
*M*_*s*_(*t*) – current straw mass [kg],
*T*_*p*_(*t*) – ambient temperature [K],
*T*_*sp*_(*t*) – exhaust temperature [K],
*T*_*sc*_(*t*) – inner walls' temperature [K],
*T*_*sc_zew*_(*t*) – outer walls' temperature [K],
*T*_*c*_(*t*) – hot water temperature (water jacket temperature) [K],
*T*_*z*_(*t*) – cold water temperature [K],
*ρ*_*sp*_(*t*) – exhaust density [kg/m^3^],
*F*_*sp*_(*t*) – exhaust mass flow to the chimney [kg/s],
*V*_*k*_(*t*) – total volume of the combustion chamber decreased by the current straw volume [m^3^],
*P*(*t*) – thermal power produced during the combustion process [W],
*F*_*p*_ – air mass flow [kg/s],
*F*_*w*_ – water mass flow through the water jacket [kg/s],
*M*_*w*_ – water jacket mass [kg],
*M*_*sc*_ – inner walls' mass [kg],
*M*_*sc_zew*_ – outer walls' mass [kg],
*α* – straw burning rate factor [1/m^2^],
*β* – convection factor of the thermal power produced during combustion,
*λ* – straw heating value [J/kg],
*ρ*_*s*_ – straw density [kg/m^3^],
*θ* – full ignition time of straw [s],
*V*_*0*_ – total volume of combustion chamber [m^3^],
*μ* – exhaust molar mass [kg/mol],
*p* – atmospheric pressure [Pa],
*R* – gas constant [J/(mol∙K)],
*c*_*p*_ – air specific heat capacity [J/(kg∙K)],
*c*_*sp*_ – exhaust specific heat capacity [J/(kg∙K)],
*c*_*sc*_ – inner and outer walls' specific heat capacity [J/(kg∙K)],
*c*_*w*_ – water specific heat capacity [J/(kg∙K)],
*K*_*s_sc*_ – factor of heat transfer between the combustion chamber and the boiler's inner walls [W/K],
*K*_*w_sc*_ – factor of heat transfer between the water jacket and the boiler's inner walls (also between the water jacket and the boiler's outer walls) [W/K],
*K*_*p_sc_zew*_ – factor of heat transfer between the boiler's outer walls and the ambient air [W/K].

Chemical reactions take place in the combustion chamber where a part of the supplied air is mixed with the straw combustion products. At any moment of time *t*, the mass balance in the combustion chamber can be described in Eq. (3). In the combustion chamber, both *ρ*_*sp*_(*t*) and *Fsp*(*t*) refer to gases that occupy the volume *Vk*(*t*). In turn, *Vk*(*t*) is the whole combustion chamber volume *V0* minus the volume *Ms*(*t*)/*ρ*_*s*_(*t*) of the currently burning straw. The Eq. (4) describes the thermal power balance in the combustion chamber. The Eqs. (5), (6), and (7) describe the thermal power balance in the inner walls, water jacket and the outer walls of the boiler, respectively. The hot water temperature is also the water jacket temperature. The Eq. (8) results from the ideal gas law (Clapeyron's equation), where the number of moles is *V*_*k*_(*t*)*ρ*_*sp*_(*t*)/*μ*. It has been assumed that the straw combustion products are an ideal gas. Since the exhaust gas is discharged freely into the chimney, the pressure inside the combustion chamber is slightly higher than the atmospheric pressure. For the purposes of the model, it can be assumed that this pressure is equal to the atmospheric pressure.

Therefore, it is assumed that a thermodynamic isobaric process occurs in the combustion chamber. The Eq. (9) describes a part of the combustion chamber volume that is filled with gas – products of straw combustion. The Eq. (10) is a slightly transformed Eq. (2). Additionally, in this equation, the Heaviside step function has been introduced with the *θ* constant. Function 1 (*t* ˗ *θ*) = 0 for *t* ˗ *θ* < 0 and 1 (*t* ˗ *θ*) = 1 for *t* ˗ *θ* ≥ 0. Thus, for *t* < *θ Ms*(*t*) = *Ms* (0), but for *t* ≥ *θ* a mathematical description of straw burning process like (2) has been given with an initial value of straw mass *Ms*(*θ*). According to (10), the straw mass begins to decrease from *t* = *θ*. The time interval [0, *θ*] is interpreted as the time interval required for the full ignition of straw. For the purpose of further analysis, it has been assumed that the air mass flow *Fp* is constant in time. Eq. (11) determines the thermal power produced during the combustion process.

The buffer tank can be also described by Eqs. (12), (13), (14), (15), and (16). The cold water temperature is also the temperature of the lowest layer of the buffer tank. It is also a lumped-parameter system. Similarly, it has been assumed that the temperatures in particular regions are uniform; in this case, however, the regions were designated in a more abstract way. Although the buffer tank physically constitutes a single unit, it has been arbitrarily divided into 5 horizontal layers (Figure 1). This model assumes that heat is transferred by conduction (e.g. between adjacent layers) and convection (e.g. a particular layer – ambient air).

(12-16) $$\left\{ \begin{array}{l} Mc_{w}\frac{d}{dt}T_{1}\left(t\right)=F_{w}c_{w}\left(T_{c}\left(t\right)-T_{1}\left(t\right)\right)+K_{1}\left(T_{p}\left(t\right)-T_{1}\left(t\right)\right)+K_{6}\left(T_{2}\left(t\right)-T_{1}\left(t\right)\right)\\ Mc_{w}\frac{d}{dt}T_{2}\left(t\right)=F_{w}c_{w}\left(T_{1}\left(t\right)-T_{2}\left(t\right)\right)+K_{2}\left(T_{p}\left(t\right)-T_{2}\left(t\right)\right)+K_{6}\left(T_{1}\left(t\right)-T_{2}\left(t\right)\right)+K_{6}\left(T_{3}\left(t\right)-T_{2}\left(t\right)\right)\\ Mc_{w}\frac{d}{dt}T_{3}\left(t\right)=F_{w}c_{w}\left(T_{2}\left(t\right)-T_{3}\left(t\right)\right)+K_{3}\left(T_{p}\left(t\right)-T_{3}\left(t\right)\right)+K_{6}\left(T_{2}\left(t\right)-T_{3}\left(t\right)\right)+K_{6}\left(T_{4}\left(t\right)-T_{3}\left(t\right)\right)\\ Mc_{w}\frac{d}{dt}T_{4}\left(t\right)=F_{w}c_{w}\left(T_{3}\left(t\right)-T_{4}\left(t\right)\right)+K_{4}\left(T_{p}\left(t\right)-T_{4}\left(t\right)\right)+K_{6}\left(T_{3}\left(t\right)-T_{4}\left(t\right)\right)+K_{6}\left(T_{z}\left(t\right)-T_{4}\left(t\right)\right)\\ Mc_{w}\frac{d}{dt}T_{z}\left(t\right)=F_{w}c_{w}\left(T_{4}\left(t\right)-T_{z}\left(t\right)\right)+K_{5}\left(T_{p}\left(t\right)-T_{z}\left(t\right)\right)+K_{6}\left(T_{4}\left(t\right)-T_{z}\left(t\right)\right) \end{array}\right.$$

where:

*t* – time [s],
*T*_*c*_(*t*) – hot water temperature [K],
*T*_*z*_(*t*) – cold water temperature (temperature of the lowest layer of the buffer tank) [K],
*T*_*p*_(*t*) – ambient temperature [K],
*T*_*1*_(*t*), *T*_*2*_(*t*), *T*_*3*_(*t*), *T*_*4*_(*t*) – water temperature in individual layers [K],
*F*_*w*_ – water mass flow through the buffer tank [kg/s],
*c*_*w*_ – specific heat capacity of water [J/(kg∙K)],
*M* – water mass in each layer [kg],
*K*_*1*_, *K*_*2*_, *K*_*3*_, *K*_*4*_, *K*_*5*_ – factor of heat transfers between a particular layer and the ambient air [W/K],
*K*_*6*_ – factor of heat transfer between adjacent layers [W/K].

**Figure 3:**
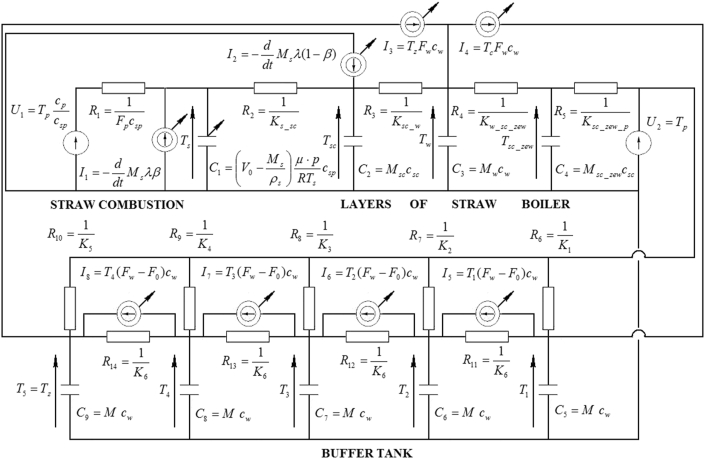
Diagram of the batch-fired straw boiler installation exhibited in the form of electrical network. Some elements of the circuit are time-variant (marked with a diagonal arrow).

Mathematical models can sometimes be presented in the form of an electrical network [27, 28, 29]. Figure 3 shows the static and dynamic properties of the installation of the batch-fired straw boiler in such a manner. In this circuit, the electrical variables have been expressed by variables of Eqs. (3), (4), (5), (6), (7), (8), (9), (10), (11), (12), (13), (14), (15), and (16). The mathematical model of the installation has been implemented in MATLAB/Simulink.

## 4 The identification of model parameters

Actual systems are usually characterized by a large number of parameters and the identification thereof is often difficult [30, 31, 32]. Two experiments consisting in straw combustion were performed using the analyzed installation in order to identify 10 unknown values of model parameters. During these experiments, the hot and cold water temperatures *T*_*c*_(*t*) and *T*_*z*_(*t*) were recorded. The identified parameter values (optimal values) recorded in case of the model and the real experiments fulfilled the condition of similarity of the hot and cold water time responses. In both the experiments the same straw mass was fed, however the combustion processes were characterized by different initial conditions. Also, in both combustion experiments the air mass flow remained constant in time, but in the first experiment it was 2 times higher than in the second one. The accurate measurement of the air mass flow was not possible and thus it was identified as one of the ten parameters. The following parameters were identified:

*F*_*p*_ – air mass flow [kg/s],
*θ* – straw full ignition time [s],
*α* – straw burning rate factor [1/m^2^],
*β* – convection factor of the thermal power produced during combustion,
*K*_*s_sc*_ – factor of heat transfer between the combustion chamber and the boiler's inner walls [W/K],
*K*_*w_sc*_ – factor of heat transfer between the water jacket and the boiler's inner walls (also between the water jacket and the boiler's outer walls) [W/K],
*K*_*p_sc_zew*_ – factor of heat transfer between the boiler's outer walls and the ambient air [W/K],
*K*_*1*_ = *K*_*5*_, *K*_*2*_ = *K*_*3*_ = *K*_*4*_ – factor of heat transfers between a particular layer and the ambient air [W/K],
*K*_*6*_ – factor of heat transfer between adjacent layers [W/K].

The identification process of the 10 parameters was difficult and computationally complex. The problem was solved numerically by using the MATLAB software. The identified (optimal) parameters minimize the performance index, which is a discrete form of the Integral Square Error. In this case, the performance index was the sum of 2 components of the expression on the right side of formula (17). Each component was the sum of the 2 expressions concerning the time responses of the hot and cold water temperature for the particular combustion experiment (18).

(17) $$Q\left(b_{1},b_{2},p\right)=Q_{1}\left(b_{1},p\right)+Q_{2}\left(b_{2},p\right)$$

(18) $$Q_{k}\left(b_{k},p\right)=\sum_{i=1}^{N}{\left(C_{k}\left(i\right)-c\left(i,b_{k},p\right)\right)}^{2}+\sum_{i=1}^{N}{\left(Z_{k}\left(i\right)-z\left(i,b_{k},p\right)\right)}^{2}$$

where:

*k* – experiment number (I or II),
*i* – time sample index,
*N* – number of time samples,
*b*_*k*_ – initial condition vector (in the model) of the *k*-th experiment,
*p* – vector of the model parameters,
*C*_*k*_ – the hot water temperature time response of the *k*-th experiment,
*c* – the hot water temperature time response of the model,
*Z*_*k*_ – the cold water temperature time response of the *k*-th experiment,
*z* – the cold water temperature time response of the model.

**Figure 4:**
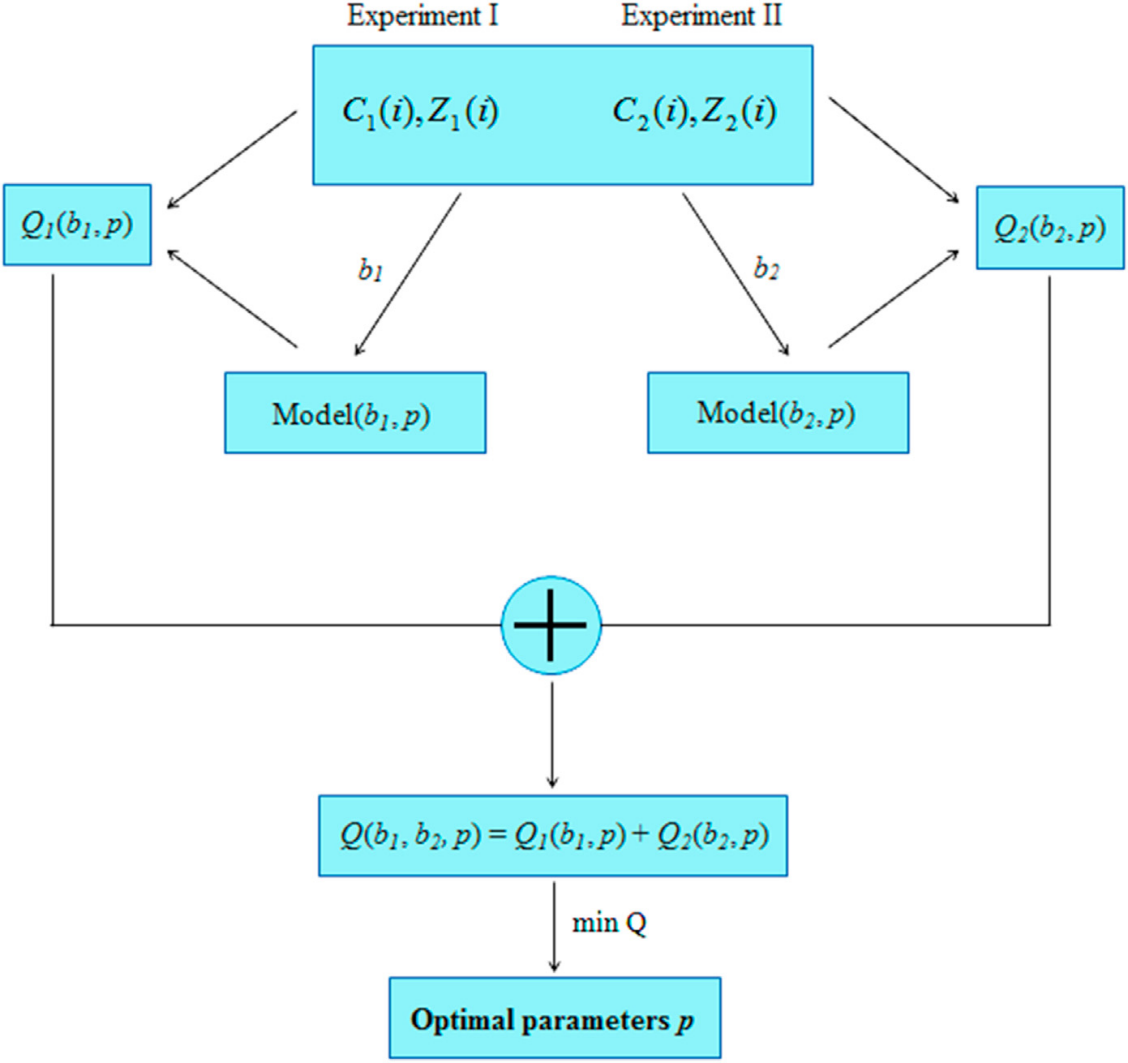
Block scheme of the parameter identification algorithm.

Figure 4 shows the parameter identification algorithm diagram.

**Figure 5:**
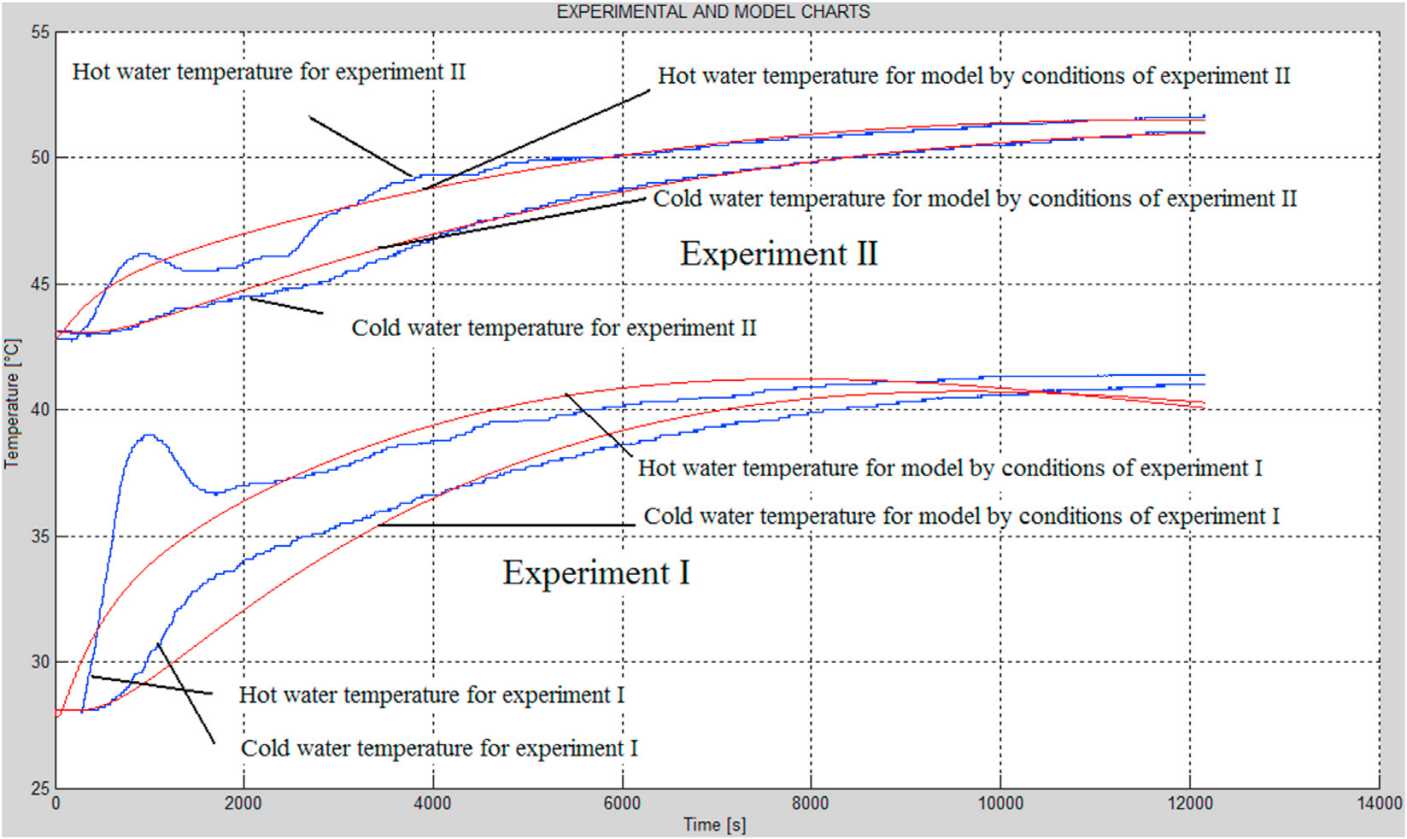
Time responses of the hot and cold water temperatures registered during the combustion experiments (blue curves) and corresponding optimized time responses of the model (red curves).

Table 1 exhibits the identification results. Figure 5 shows the time responses of the hot and cold water temperatures registered during the combustion experiments (blue curves) and the corresponding optimized time responses of the model (red curves).

**Table 1:** Parameters identified based on the model and two combustion processes.

No.	Parameter	Optimal value
1.	*F*_*p*_	0.0277 [kg/s]
2.	*θ*	1.0600 [s]
3.	*α*	0.1373 [1/m^2^]
4.	*β*	0.3972
5.	*K*_*s_sc*_	0.0000 [W/K]
6.	*K*_*w_sc*_	1200000 [W/K]
7.	*K*_*p_sc_zew*_	241.6433 [W/K]
8.	*K*_*6*_	50003 [W/K]
9.	*K*_*1*_	61.9391 [W/K]
10.	*K*_*2*_	0.0000 [W/K]
11.	**Performance index**	**32031**

Despite the optimization of the model parameters, significant discrepancies may be noted between the model and the experiments. The differences likely arise from the fact that the thermal processes occurring in the installation are more complex. Probably, the discrepancies between the model and the experiments have been mostly influenced by the phenomena occurring in the actual straw combustion processes.

Figure 5 clearly shows that the hot water temperature time responses in the experiments (especially in the first experiment) has a significant local maximum at the initial part of the analyzed time interval. A similar maximum can be noted after the same time in the second experiment, it is, however, less distinct. In similar curves pertaining to the model, these effects are not observed.

In the opinion of the author, these discrepancies may be explained by the change of the straw burning rate factor over time. To perform this analysis, one should define a momentary straw burning rate factor, understood as the straw burning rate factor for a thin straw layer on the currently burning surface of the straw cube. The hot water temperature curves pertaining to the experiments may be interpreted in such a manner that in the early part of the analyzed time interval the straw is burning much faster than it would result from the model in consideration. This can be explained by the fact that straw is drier at the edges than deeper towards the center, therefore the edges of the fed cube exhibit a greater burning rate factor. In case of the second experiment, one can observe a rapid growth of the straw burning rate factor later in the analyzed interval, followed by a slow decrease.

**Figure 6:**
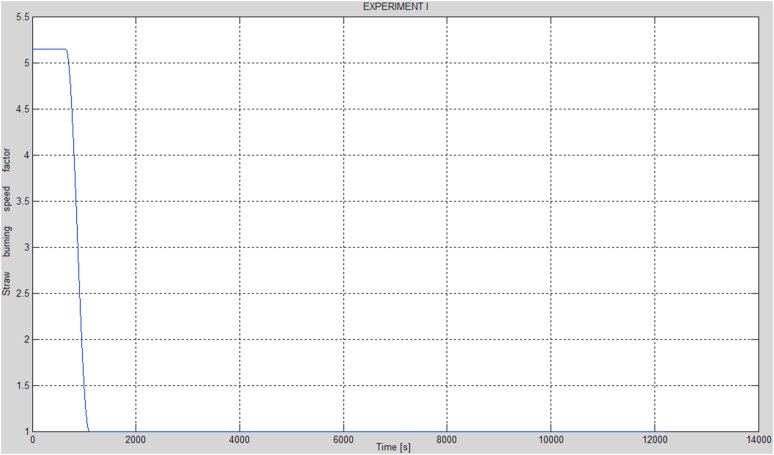
Curve of the momentary straw burning rate factor in relation to its basic model value for the case of the first experiment.

**Figure 7:**
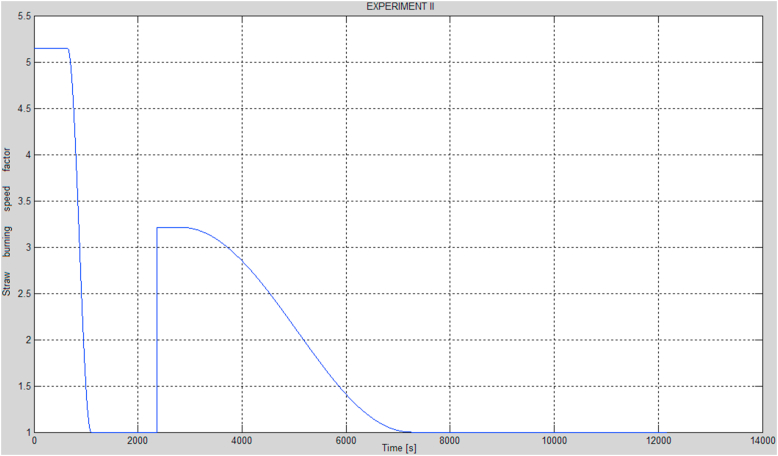
Curve of the momentary straw burning rate factor in relation to its basic model value for the case of the second experiment.

**Figure 8:**
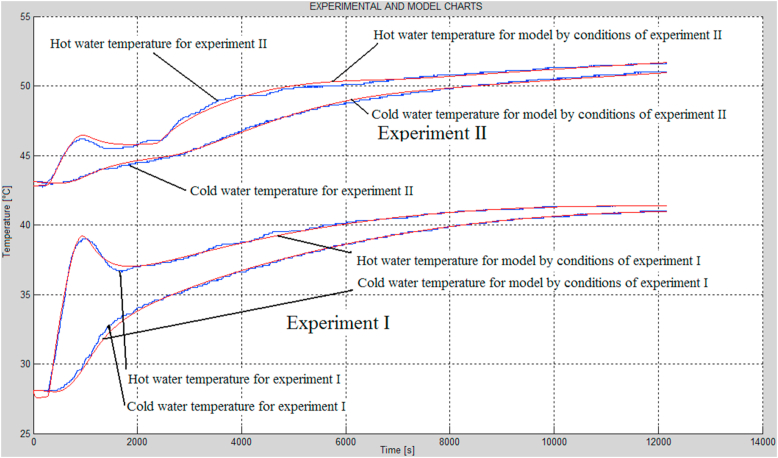
Time responses of the hot and cold water temperatures registered during the combustion experiments (blue curves) and the corresponding time responses exhibited by the modified model, determined by optimization (red curves).

Assuming the curves of the momentary straw burning rate factor in relation to its basic model value are as presented in Figure 6 and Figure 7, the red curves in Figure 8 would represent the optimized parameters of the modified model, the time responses of the hot and cold water temperatures. The curves of the momentary straw burning rate factors and the previously mentioned parameters have been obtained by optimization. In addition, it has been assumed that the decreases of the momentary straw burning rate factors (presented in Figure 6 and Figure 7) assume a shape similar to the one between the maximum value and the nearest minimum value of the *cosine* function, as provided by formula (19). An important advantage of the function (19) is that the change between the 2 extreme values proceeds in a continuous and smooth manner, possible in case of actual combustion processes. On the other hand, a rapid growth of the momentary straw burning rate factor for the case of the second experiment has been explained as an instant change of the form of straw. This situation may occur when a part of the burning straw rolls over, thereby significantly increasing the burning surface area, resulting in a higher combustion rate.

(19) $$f\left(t\right)=\left\{ \begin{array}{c} P,t<t_{min}\\ \frac{cos\left(\frac{\pi \left(t-t_{min}\right)}{t_{max}-t_{min}}\right)+1}{2}\left(P-1\right)+1,t\in \left[t_{min},t_{max}\right]\\ 1,t>t_{max} \end{array}\right.$$

The values of the parameters described in formula (19), such as *P*, *t*_*min*_, *t*_*max*_ were calculated by means of the MATLAB software and curves presented in Figure 6 and Figure 7 were generated for these values. All the optimal parameters have been presented in Table 2.

**Table 2:** Parameters identified based on the modified model and the two combustion processes.

No.	Parameter	Optimal value
1.	*F*_*p*_	0.0064 [kg/s]
2.	*θ*	287.6186 [s]
3.	*α*	0.4654 [1/m^2^]
4.	*β*	0.6579
5.	*K*_*s_sc*_	0.4107 [W/K]
6.	*K*_*w_sc*_	1199600 [W/K]
7.	*K*_*p_sc_zew*_	1.3603 [W/K]
8.	*K*_*6*_	59999 [W/K]
9.	*K*_*1*_	0.0204 [W/K]
10.	*K*_*2*_	40.3320 [W/K]
11.	*P* (experiments I and II)	5.1497
12.	*t*_*min*_ (experiments I and II)	659 [s]
13.	*t*_*max*_ (experiments I and II)	1113 [s]
14.	*P’* (experiment II)	3.2127
15.	*t*_*wz*_ (experiment II)	2363 [s]
16.	*t*_*min*_*’* (experiment II)	2977 [s]
17.	*t*_*max*_*’* (experiment II)	7295 [s]
18.	**Performance index**	**1038**

where:

*P* – initial value of the momentary straw burning rate factor in relation to its basic model value in case of both experiments,

*t*_*min*_ – time of the beginning of the decrease of the initial value of the momentary straw burning rate factor in relation to its basic model value in case of both experiments [s],

*t*_*max*_ – time of the end of the decrease of the initial value of the momentary straw burning rate factor in relation to its basic model value in case of both experiments [s],

*P’* – the growth of the value of the momentary straw burning rate factor in relation to its basic model value in case of experiment II,

*t*_*wz*_ – the time of the growth of the momentary straw burning rate factor in relation to its basic model value in case of experiment II [s],

*t*_*min*_*’* – time of the beginning of the decrease following the growth of the value of the momentary straw burning rate factor in relation to its basic model value in case of experiment II [s],

*t*_*max*_*’* – time of the end of the decrease following the growth of the value of the momentary straw burning rate factor in relation to its basic model value in case of experiment II [s].

The values of the parameters in Table 2 are much more reliable than those obtained in case of Table 1, as in the case of the modified model, the performance index was much lower. These considerations exhibit that there is a significant similarity of the temperatures of hot and cold water when the modified model and the combustion experiments are compared (Figure 8). It should be noted, however, that the modified model and the basic model are in fact the same model, and they differ from each other only in terms of the modeling of straw combustion. The kind of the straw combustion is only the result of the type of straw. Assuming that the straw burning rate factor changes over time, it is possible to achieve nearly perfect overlapping of the time responses of the model and the experiments.

## 5 Conclusions

This study presents a specification of the straw combustion in a batch-fired straw boiler without consideration to the chemical processes.

The description of other phenomena taking place in the straw boiler and the buffer tank has been given by means of ordinary differential equations. In spite of the optimally determined model parameters, significant discrepancies between the model and the experiments have been observed. These discrepancies have been largely eliminated by the modification of the model of the combustion process. Assuming the model of the straw combustion has been modified, the remaining parts of the entire installation do not require any adjustment. The initial model of the straw combustion process also seems to be justified – only under the assumption, however, that the loaded straw exhibits the same burning rate factor during the entire combustion time and the combustion process proceeds without any significant interference.

The results of the paper validate the statement that in order to achieve a better convergence of the original model with experimental data, one should take a closer look at the straw combustion process, especially in the context of the variable straw burning rate factor. Straw intended to be used as fuel for the straw boiler may become much drier at the edges than towards the inside during storage and thus it may initially exhibit a higher burning rate factor. During the second experiment, a part of burning straw block could have separated and rolled over, resulting in a much higher burning surface area and thus a much higher straw burning rate factor.

Owing to the modified model, the changes of thermal power consumption during the combustion of the straw load may be analyzed depending on the installation operating parameters. The results of the model may be applied in the optimization of the configuration of the entire installation.

## Declarations

### Author contribution statement

Wojciech Kreft: Conceived and designed the experiments; Performed the experiments; Analyzed and interpreted the data; Contributed reagents, materials, analysis tools or data; Wrote the paper.

### Funding statement

This work was supported by the Polish 10.13039/501100004569Ministry of Science and Higher Education (agreement no 11.11.120.396).

### Data availability statement

Data included in article/supplementary material/referenced in article.

### Declaration of interests statement

The author declares no conflict of interest

### Additional information

No additional information is available for this paper.
